# Hospital and Institutional News

**Published:** 1916-02-26

**Authors:** 


### HOSPITAL AND INSTITUTIONAL NEWS.

### THE FATHER IN OUR KING.

King George, by continuous and devoted personal service, has acquired a sound knowledge of the hospital atmosphere. This week, in one of his visits to a hospital, he encountered a small boy aged four who had dropped his toy, and appealed to the King to "pick up the injun." This the King laughingly did and won the boy's confidence. When the King was passing to the next cot the small boy called him back to see his picture book. His Majesty immediately complied with the child's wish, and entered into his pleasure as he exhibited his favourite pictures. How striking is the contrast in ideals revealed by King George with the baby patient and the Kaiser with his cultured baby-killing policy!

### ST. GEORGE'S HOSPITAL.

Lord Newton has been selected to fill the vacancy in the joint treasurership of St. George's Hospital, due to the resignation, through serious illness, of Sir Gerard Lowther. Lord Newton has accepted the invitation, and we congratulate the authorities upon their successful action.

### SIR WILLIAM TURNER.

The death of the Principal and Vice-Chancellor of Edinburgh University at the ripe age of eighty-four removes one of the best organisers and greatest men the medical profession produced in the last century. The late Principal was Professor of Anatomy at Edinburgh for thirty-six years, and had been connected officially with the University there for more than sixty years. He had a brilliant academic career (at St. Bartholomew's Hospital), and was early marked down at Edinburgh as a man of commanding intellect and force of character. As a lecturer and professor his fame was world-wide; but he was equally great at the statecraft of University government and at problems of business and finance. He was President of the General Medical Council from 1898 to 1904, and President of the British Association in 1900. Amidst all his other interests, he made time to be an ardent Volunteer for thirty-one years, retiring with the rank of a lieutenant-colonel and the V.D. Although so long and so closely connected with Edinburgh, Sir William Turner was not a Scot, but a Lancashire man. He was knighted in 1886, and made K.C.B. in 1901. He leaves three sons, two of whom are in the medical profession: one is a well-known London nerve specialist, while the other practises as a throat and nose surgeon in Edinburgh.

### A CANVAS-CAMP JOURNAL FROM MALTA.

We have received the first number of the *Ghain Tuffieha Record*, the journal of the camp of that name which has been established in Malta. Edited by Colonel Maurice, C.M.G., R.A.M.C., the officer in command, the *Record* relates the arrival in August last of No. 17 Stationary Hospital from England. Nearly 500 men were then in camp. No. 17 then started tent-pitching on a large scale, and in spite of the inexperience of the staff, who, we learn, were all New Army men, three divisions of the camp—the three present hospitals—were quickly organised. In September No. 17 Stationary Hospital left for Gallipoli, leaving Colonel Maurice behind to carry on the organisation. The work, he tells us, is done almost entirely by convalescent patients, and over 10,000 men have been under his charge. He remarks that he has had "surprisingly little bother" and that, "taking them all round, the Australians have given him most help and most trouble." The *Record* has the double aim of acquainting the inhabitants of the camp with the machinery by which it is organised, and of chronicling the varied activities, humorous or otherwise, which make up the life of the place. The result is neither dry nor trivial, and no one could read the articles on "The Camp," and the bird-life of the place on the one hand, nor the accounts of the sports and debating societies on the other, without knowing that he had got his money's worth. Illustrations and poems complete the whole. Remembering the difficulties of production we heartily congratulate the Commandant on the success of his first number, which promises well for those yet to come.

### THE SQUIRE OF HAWARDEN.

After considering various schemes for the establishment of a memorial to the late Lieutenant Gladstone, the memorial committee have decided to accept the offer of the Chester Royal Infirmary, and to convert the new ophthalmic theatre, with the wards on each side of it, into a memorial. The sum so far raised is £560, and with a further £100 the architect estimates that the cost would be provided. A tablet and a portrait of the late Squire of Hawarden will be placed at the entrance of the new ward, which will be named after him. Members of the Gladstone family are placing a memorial in Hawarden Parish Church, but the committee have decided that the church shall also contain a tablet on which the gifts to the Chester Royal Infirmary will be recorded.

### ASSESSORS IN COMPENSATION CASES.

A case was recently decided at the Epsom County Court in which the services of an assessor were very opportunely utilised by the Judge; although the case was trivial in itself, there were one or two points about it which show that the assessor's report decided the verdict. Briefly, there was a conflict of evidence as to whether a stiffness of an old man's knee was or was not due to a slight accident which he had sustained about a year before. The doctors who had attended him at the Carshalton Hospital, where in the meanwhile he had been operated on for a tumour in another region, held that he had osteo-arthritis in both knees, not due to the accident; whereas a doctor from Harley Street put forward the opinion that the accident caused the subsequent stiffness of the knee. As far as can be judged from the report of the case, the basis for this last opinion was somewhat slender; but the name of Harley Street is still a power in the land, so the Judge called in an assessor, whose name is not given—possibly he also lives in the Cavendish Square district. The assessor was quite positive that the accident had nothing to do with the osteo-arthritis, and the Judge acted on that view. It is to be wished that Judges would more frequently call in an assessor when confronted, as they often are, by a conflict of medical testimony .

### PATHOLOGICAL WORK AT MAUDSLEY HOSPITAL.

The transfer of the equipment of the pathological laboratory at Claybury Mental Hospital from that institution to the Maudsley Hospital at Denmark Hill was part of the original scheme for the equipment of the latter building for use as a mental hospital. The transfer was to have been postponed in consequence of the Maudsley Hospital buildings being taken over by the Army Council for use as a war hospital, but at the request of the Army Council arrangements are now being made for the transfer to take place at once, as the laboratory equipment will be useful in connection with war hospital work. The London Mental Hospitals Committee has been assured that the equipment will continue to be available for its original functions in connection with the London County mental hospitals, and their control over it will still be maintained.

### THE LAMBETH MEDICAL BOYCOTT.

The Lambeth medical boycott has not ended, but the Borough Council has now appointed Dr. Arthur Cooke, of Kennington Park Road, hon. assistant physician at the Tuberculin Dispensary, Chelsea (formerly at Kennington), as part-time assistant medical officer of the Municipal Tuberculosis Dispensary, at a salary of £350 a year. The lady, Dr. Eleanor Gorrie, who has been acting as *locum tenens* during the boycott, was recommended for appointment, but the Council thought Dr. Cooke had greater qualification. Originally the Council proposed to appoint an assistant officer at a salary of £300, but the British Medical Association contended that the vacancy was really one for a full and not an assistant medical officer, and that the salary should accordingly be £500. The Council increased the salary to £350, but the Association still recommended its members to refrain from applying for the situation. Eventually the Council decided that the appointment should be for "part-time" at £350 a year. Nevertheless the boycott was not removed, and there were only three applications, from Dr. Gorrie, the *locum tenens*; Dr. Cooke, who has now been appointed; and Dr. Jordan. The Borough Council assumed that it would be possible to secure a doctor with a local practice, but in this connection the Local Government Board stated that difficulties might arise from the appointment of a general practitioner owing to the fact that in the course of his dispensary work and of work under the Board's General Order for the Domiciliary Treatment of Tuberculosis, he would have to deal with patients of other practitioners.

### A CASE OF LEAKAGE.

According to a paragraph in *Truth*, recently enlisted recruits under the Derby scheme have received copies of a pamphlet issued by one of the anti-vivisection societies urging them not to submit to inoculation against enteric fever, and the editor of that paper states that he is satisfied that the names and addresses have been obtained from an official list. He very rightly points out, without taking any definite line for or against inoculation, that no good purpose can be served by unsettling the minds of enlisted soldiers by urging them to make an individual stand against the considered opinion of the medical authorities. Nor should the names and addresses of recruits be obtainable by propagandists of any kind. We should have been glad to see a journal run with the common sense and intelligence ordinarily displayed by *Truth* taking a rather stronger line on the question of inoculation, and examining impartially the statistics of enteric fever as accumulated during the course of this campaign; there could be little doubt as to the verdict. But, apart from this, it must surely be plain to everyone (except the propagandists themselves) that this method of spreading their doctrines is underhand and derogatory, and that such a pamphlet is necessarily in intention subversive of discipline, and therefore of efficiency in the Army. We should be glad if this leakage of official lists were traced to its source and dealt with.

### A GOVERNMENT HOSPITAL IN PEKING.

It is announced that a hospital supported by the Chinese Government is to be established in Peking, and that a sum of 200,000 taels is to be provided for the purpose. This is a most significant step, pregnant with meaning: for hitherto, unless we are mistaken, only military hospitals have been started under Government control, though occasional assistance has been given to European (missionary) hospitals. Another indication that Western ideas are making an impression in the East is the selection of Dr. Wu Lien Teh as director of the hospital. He is, undoubtedly, the only possible selection for such a post under a Government which is in earnest over the matter: so that his appointment is pretty good evidence that this hospital is intended to be efficient, and to be a real credit to China. Dr. Lien Teh was educated in England, at Cambridge and St. Mary's, and his career since he returned to China has been one of increasing and successful endeavour to introduce the highest Western ideals into Chinese medicine.

### THE LISCARD HOSPITAL AFFAIR.

The executive committee of the Liscard Hospital has held an inquiry into the case of the woman who, as reported in The Hospital, was refused admission, and eventually died in her own home. The committee places entire responsibility on the resident house surgeon, and quotes part of an apologetic letter from him in which he refers to his "negligence" in not seeing the case. In another letter to the Press the doctor declares that the

" vital point " was that the woman left on the distinct understanding that she was to be taken to the Union hospital. In our view, however, the vital point is rather different. Even though he was in bed, we agree that the doctor should have seen the woman, but what has yet to be explained is why the nurse, apparently acting on instructions or general precedent, informed the relieving officer that the patient could not remain in the hospital until he had time to examine the circumstances himself. It is all very well for the doctor to say that the woman would " never have been allowed to leave this institution had not this provision for her comfort been made." But as " the understanding that she should be taken to the Union hospital with the consent of her family " had been come to without consulting them it was hardly " providing for her comfort." Her family refused to let her go, regarding the proposal with indignation. The relieving officer asked to see the woman in hospital before he could admit her. This, for some unexplained reason, was refused. She was turned away, which was the cause of the whole trouble. Perhaps Mr. C. R. B. Gilchrist, the chairman, will explain this vital omission in his committee's report. The matter is doubly regrettable as a memorial tablet to Nurse Cavell was unveiled only last week.

### WAR WASTAGE.

We have received a notice of the annual meeting, issued to the subscribers and donors of the Prince of Wales's General Hospital, Tottenham. The business on the agenda is purely formal. The notice and extracts from the rules occupy two pages of print, and a proxy form of unnecessarily large size is also sent. We desire to call Viscount Hill's attention to this manifest waste of paper, and to the avoidable expenditure thus incurred. Such extravagance and inutility in war-time of the procedure call for comment.

### THE HOSPITAL TREATMENT OF SICK PRISONERS OF WAR.

Inevitably, in time of war, the doings of the German Hospital, Dalston, excite a special interest, and, indeed, the past year's work is remarkable in several respects. The number of in-patients, for instance (which has fallen off from 2,108 to 1,687), included 375 sick prisoners, who had been transferred to the hospital from various internment camps. Of course, patients of other nationalities besides German have also been treated, and, owing to the War Office's acceptance of the committee's offer of their convalescent home at Hitchin, the average stay of each patient has risen to the remarkable figure of thirty-six days. (It was twenty-three in 1914.) The number of out-patients has practically been halved; and, unfortunately for the hospital, the income, both from subscriptions and donations, has been halved also. Indeed, it may be said that donations would have practically reached vanishing-point had not the treasurer issued an appeal which realised £2,447. Moreover, £100 was received from the King's Fund, and £400 accrued from legacies. The financial result at the end of the year has been a deficit of £3,800, which has been met by transfer from the deposit account. The annual meeting, which was held last week, was presided over by Baron Bruno von Schröder, the treasurer, on whom, as will have been apparent, fell the task of making an undoubtedly urgent appeal as successful as the obvious difficulties of the time permitted. The outlook can hardly be said to have improved, but we trust that the present year may prove the last in existing circumstances.

### THE ROLL OF HOSPITAL-HOUSES.

When the history of voluntary effort on behalf of the wounded during the war comes to be written, one chapter will have to concern itself with the converted country house. A very interesting document in this connection is the list of country houses lent by their owners for hospital purposes, which the Hon. Arthur Stanley, chairman of the joint committee of the British Red Cross Society and the Order of St. John of Jerusalem in England, has supplied. The list is comprehensive, and includes country houses thus lent, whether or not in their owners' absence; town houses converted into hospitals; and houses lent directly to the Red Cross Society. The houses are classified under the names of the counties in which they are situated; the owner's name and the number of beds are given in each case, together with an indication as to whether each house is or is not a V.A.D. hospital. This roll of " hospital-houses," to use a convenient term, is of interest from many points of view, and forms a remarkable record in itself.

### A HOSPITAL SECRETARY AMONG THE POETS.

The series of articles on " Hospitals and the Poets " have dealt mainly, as our contributor has pointed out, with great poets either engaged in a medical training, like Keats, Goldsmith, or Crabbe, or with a man of letters, like Henley, who described in his poems the patient's point of view. As a footnote to these articles it is pleasant to recall that among our contemporaries one hospital secretary at least has published several volumes of verse. Mr. B. Burford Rawlings, the former well-known secretary of the National Hospital, Queen Square —whose *Hospital in the Making* is as much a critical study of hospital questions as a contribution to hospital history—has also published a drama in blank verse entitled *A Story of Unrest*, and two collections of verse entitled *The Waters of Ar'gyra; The Vision of Belshazzar* and *The Wooing of a Goddess*. This is sufficient to show that while a poet may write of hospitals, a man who has made hospital work and administration his main preoccupation may also write verse. But, speaking from memory, we fancy that Mr. Burford Rawlings has kept the two spheres of hospital life and poetry quite distinct. He has rarely, if ever, attempted to marry them. This is a pity, for until the two are married we shall still have to write of hospitals and the poets as if the two were naturally opposed to one another. Henley and Walt Whitman have proved the contrary.

### PUBLIC LIBRARIES AND HOSPITAL PATIENTS: A NEW SCHEME.

A new scheme for the supply of books to the patients is being started at the Coventry and Warwickshire Hospital. In view of the haphazard way in which hospital libraries for the benefit of patients are usually organised, it may be helpful to publish a general outline of the scheme. The idea at the back of it emanated apparently from the City Librarian, who suggested that it might be possible to supply parcels of books for the patients, if someone would undertake responsibility for the safety of the volumes. This, we understand, the matron has kindly done; and a library is being started which should have the double advantage of being far more varied and extensive than any which most hospitals could afford to purchase, or even accommodate, and of being kept up to date by a constant change of books. It is at once evident that the basis of the new scheme is co-operation between the public library of the city and some member of the hospital's staff—in this case the matron—who will accept responsibility for the safety of the books. What is possible at Coventry should be possible wherever a hospital and a public library co-exist in the same town; and the plan appears to offer the simplest and most effective remedy for the wretched supply of books to which most hospital patients are limited.

### A NEW PROPOSAL FOR REGULATING STREET COLLECTIONS.

Mr. J. Carlton Stitt, who presided at the Liverpool Hahnemann Hospital's annual meeting last week, complained that the institution had not benefited by any of the recent street collections, although sick and wounded soldiers had been received for treatment. He understood that Liverpool had decided to apply for powers to regulate street collections, so that the conditions under which any were permitted could be controlled by the terms of the licence authorising them. In allusion to the regulations made by the Commissioner of the Metropolitan Police, with the approval of the Home Secretary, under which, by rule 9, the Commissioner might grant a permit only after he had received a certificate from an advisory committee (appointed by him and officially approved), Mr. Carlton Stitt said: "The executive committee of the Liverpool Council of Voluntary Aid is preparing a special memorandum, to be laid before a conference, and I suggest that while the Council of Voluntary Aid should be represented on any advisory committee which may be formed here on the London model, the applicant for a permit to hold a street collection should also, and at the same time, present a scheme of distribution of the money so raised." This searching proposal was received with applause; and the fact that it may have been inspired by a sense of grievance need not blind our eyes to its practical force. Street collections of late have brought in so much money, so quickly, that there has been a tendency to glorify the result, and let the spending take care of itself, in the lackadaisical belief that there will be enough cash for everyone to silence criticism. To prevent street collections from defeating their object by becoming a public nuisance they must be limited in number, and that they may be so limited they must be controlled. Why not in expenditure as well as in organisation? Can this be achieved without squabbles over the expected "swag" on the part of interested institutions?

### THE TRAMWAY COLLECTIONS IN MANCHESTER.

Hospital Sunday is over at Manchester, and the collections in the churches have been supplemented by a full week's contributions in the boxes on all the tramcars of the city. A correspondent assures us that it would be an excellent thing if, in the years to come, the disused ticket-boxes on the cars could be used occasionally, as they have been throughout the war, for donations for charitable objects. Up to the present fifteen or twenty thousand pounds have been raised in this way in Manchester, and the cars bear prominent announcements week by week as to the particular charity which is to benefit by the contributions. The hospitals, the British Red Cross Society, and the nursing profession have already benefited substantially from tramcar collections, and there can be little doubt that many persons—non-churchgoers and others—contributed to the Hospital Sunday effort who would otherwise have been missed by the ordinary appeals. The plan, which has proved so successful at Manchester, deserves to be known elsewhere.

### A USEFUL COMMITTEE.

The Leicester Royal Infirmary Matinée Committee has rendered exceptionally useful service since its formation some years since. The past few months have, however, demonstrated the usefulness of a permanent organisation ready at short notice to take up efforts which circumstances and time may render desirable. Three times since the war the committee has arranged matinées to wounded soldiers from the hospitals of Leicestershire, and 1,000 men were this week privileged to enjoy a special programme which, under Mr. Oswald Stoll's approval, had been arranged. By the courtesy of the members of the Leicester Automobile Club, the wounded men were conveyed to and from the matinée by motor-cars lent for the occasion. On arrival they found reserved seats, and a supply of cigarettes, chocolates, etc., provided by the Matinée Committee. In thanking the artistes and all who had assisted in giving joy and pleasure to the wounded, the Mayor (Alderman North) indicated his approval of the work of the committee by announcing that he would act as president of the Matinée Committee for the year, and that the annual effort for the infirmary would, with Mr. Oswald Stoll's permission, be held on April 27 next.

### THE COMPUTATION OF SUPERANNUATION ALLOWANCES.

A recent decision of the Local Government Board upon the method of computation of superannuation allowances will be of interest to the many Poor-Law medical officers who hold more than one office under a Board of Guardians. The clerk to the Maidstone Guardians, at the date of his resignation, had held offices under the Poor Law for more than forty years. During the last five years of his official life he had held the post of registrar of births, deaths, and marriages, as well as his clerkship. The point in dispute was whether he was entitled to his superannuation allowance at the rate of forty-sixtieths of the salaries of the combined offices, or whether the allowance should be calculated as forty-sixtieths of his salary as clerk and five-sixtieths of his salary as registrar. The Local Government Board, after referring the question to the Law officers of the Crown, has decided that in such cases the retiring officer is entitled to a superannuation allowance calculated on the average amount of his combined salary for the five years preceding his resignation at the rate of one-sixtieth for every completed year of his total service under the Poor Law up to a maximum of forty-sixtieths.

### THE MEDICAL STAFF AT KINGSTON POOR-LAW INFIRMARY.

THE method of appointing the junior medical officers at the Kingston Infirmary was discussed at the last meeting of the Guardians. The system at present is that the medical superintendent appoints and the Guardians confirm the appointment. Many of the Guardians were of opinion that the Board should see the selected candidates and make the appointment themselves. The latter is the procedure in vogue at most Poor-Law infirmaries. There can be no doubt, however, that the medical superintendent is the man best qualified to select the most suitable candidate, and the Guardians decided to continue their present practice. During the discussion some of the Guardians protested against the appointment of a third medical officer at the infirmary. They considered that a medical staff of two should be sufficient. This is interesting as showing how little importance many Guardians attach to the provision of adequate medical attendance. The infirmary contains 500 patients and the medical staff consists of a part-time medical superintendent and two assistant medical officers. Anyone acquainted with hospital work would say that, if all these patients are really suitable for treatment in an infirmary, the medical staff instead of being too large is too small. Either the patients are not receiving the medical attention they need, or a large number of them do not need much treatment and would be more suitably placed in less expensively equipped institutions.

### WRAPPING-PAPER FOR FOOD.

AN inquiry into the source and cleanliness of the paper used for wrapping up articles of food intended to be eaten without cooking or washing is one of more than passing interest from the point of view of public health. Such an inquiry was undertaken in the Borough of Finsbury, and the results are reported by Dr. A. E. Thomas, the medical officer of health. The premises concerned were shops in slums and street stalls, and included those selling fried fish, ham, hot puddings, groceries, sweets, and so forth. The paper used consisted, as might be supposed, chiefly of newspapers and pages of magazines, but old time-tables and directories contributed their quota. On the whole the papers examined were clean, but exceptions were not rare. Storage places were often open to severe criticism, any odd corner, damp or dusty, having to serve for the purpose. As for the sources of the bulk of the paper, these were mostly free from objection, such as dining-rooms, printing establishments, offices and proprietors of patent medicines, the last presumably using the demand as a medium for advertising. Fried-fish shops obtain their paper from a special dealer well known to the trade. Some shopkeepers obtained their supply from neighbouring householders, but without inquiry as to the presence of illness in the house. The result of the inquiry was to disclose no gross crimes against sanitary law, and as fastidious persons do not commonly obtain food in this fashion, their harrowed feelings may be left out of account.

### THE INDISPENSABLE ATTENDANT.

IT is interesting to learn from the report of the visitors of the Gloucestershire mental hospitals that since the outbreak of war 44 per cent. of the attendants have left institutional life for active service. Even the remainder, it appears, so far as age and health allow them, have been attested and grouped in Section B Army Reserve. The places of the enlisted men have been filled to some extent by men who are either over military age or else have been rejected. But a sufficient number has not been found to fill all the vacancies, and in view of the need for retaining at least two active and experienced attendants in the wards for acute cases, the Local Tribunal is to be requested to postpone calling up some of the men. Difficulties, moreover, have been increased for everyone by the temporary overcrowding of the institutions through the reception of some of those patients who were displaced when Cardiff Mental Hospital became a military hospital. That, then, it may be necessary to retain a few at least of the "eligible" attendants is obvious, and the Local Tribunal will, no doubt, fully realise the need.

### IMPORTANT ALTERATION IN THE POISON SCHEDULE.

THE Council of the Pharmaceutical Society, in pursuance of their powers under the Pharmacy Acts, have made an important alteration in the Poison Schedule, the effect of which is to transfer to the first part of the Schedule all preparations of opium containing 0.75 per cent. of morphine. This has been done with the specific object of placing the sale of laudanum prepared according to the British Pharmacopœia of 1898 under the same restrictions as the laudanum of the new Pharmacopœia. It will be remembered that the Pharmacopœia of 1914 raised the morphine content of tincture of opium from 0.75 to 1 per cent., and the result of this was that while the stronger preparation could only be sold by chemists to persons known to them, the purchaser being required to sign the Poison Book, the old-fashioned "laudanum" could continue to be sold without these restrictions. The affair was further complicated by the fact that if a purchaser demanded laudanum it was a legal obligation on the pharmacist to supply the tincture of the new Pharmacopœia, although the purchaser in most cases would expect to get the weaker preparation. Obviously this condition of things was open to serious objection; but it is equally clear that the resolution of the Pharmaceutical Society which places the old laudanum in the first part of the Poison Schedule does nothing to alter it. It will still happen that people will be supplied with the stronger preparation when they want the weaker one, but, at the same time, it is all to the good that difficulties should be put in the way of the purchase of laudanum.

### THE AMERICAN WOMEN'S WAR HOSPITAL.

It will be within the recollection of most of our readers that at the beginning of October the two hospital units detailed for service in Great Britain by the American Red Cross Society were recalled to the United States after one year's service, owing to the exhaustion of the funds provided for the purpose. Two of the American surgeons, Drs. Penhallow and Daniel, remained at the hospital, which was taken over by the American Women's War Relief Fund and named the American Women's Hospital. The hospital, which is at Paignton, South Devon, is now under a military commandant (Lieut.-Colonel Gunning, R.A.M.C.), and its work has continued absolutely unhindered by the change of management. As evidence of this may be cited the report on the second thousand cases admitted, just submitted to the executive committee by the chief surgeon, Dr. Penhallow. It covers the period from April 3 to December 8, 1915, and thus includes cases both before and after the change was made. The report is mainly statistical, and it is evident that good work is being done.

### A DEMAND FOR HOSPITAL BEDS FOR ADVANCED CONSUMPTIVES.

The importance of providing accommodation for advanced infectious cases of consumption is being realised by the Insurance Committee for West Sussex. At a recent meeting of the Sanatorium Benefit Sub-Committee, a recommendation was adopted which expressed concern at the number of advanced cases of pulmonary tuberculosis which came before their notice, and the County Council was urged to adopt measures at once for the segregation of infectious cases. One of the speakers criticised severely the County Council for failing to provide the necessary hospital accommodation, and he quoted a statement by Mr. Lloyd George to the effect that capital grants will continue to be made for sanatoria where necessary, and that local authorities will not be prohibited from borrowing for this purpose. This was, in the speaker's opinion, an answer to the excuse of the County Council—namely, that owing to the war a loan could not be obtained. It was alleged that the inaction of the County Council was due to want of sympathy on the part of some of its members with the Insurance Act. All tuberculosis workers will admit that segregation of advanced cases is a vital factor in combating tuberculosis, and is a measure which will repay the expense.

### THIS WEEK'S DRUG MARKET.

Possibly for the reason that the prices of many drugs have reached the limit beyond which the demand for them would be reduced to a minimum, the general advancing tendency in market values is much less pronounced than it was a short time ago. Indeed, since the beginning of the year such remarkable instances of sharp upward movements as were quite a common experience last year have been rare. Nevertheless the drug market is in a very curious position, and it is more than ever desirable that buyers should exercise great caution and make full inquiry into the market condition of all drugs they propose to purchase. In synthetic drugs there has been no noteworthy advance in price since the beginning of the week; on the contrary, the high prices of several of them are not quite so firmly maintained as they were. Thus the downward movement in the price of hexamine which began a few weeks ago has continued, and supplies of several other synthetic products are offered a little more freely than they were, although there have been few, if indeed any, quotable changes in prices. Chloral hydrate can be bought on slightly better terms. Citric acid is again dearer, and prices of cream of tartar and tartaric acid have a distinctly upward tendency. Potassium chlorate is much dearer, and potassium permanganate has experienced a further advance in price. Quotations for new season's cod-liver oil come substantially higher than the prices recently ruling for last season's oil, but it is still impossible to form any reliable opinion as to the future of this market. A further sharp advance has taken place in the price of lycopodium. The high value of opium is well maintained, and there is no change in the position of bromides. Holders of Continental brands of quinine having relaxed somewhat in their views, a considerable business has been done at rather lower prices, but any substantial reduction is hardly to be expected.

